# Diagnostic and Therapeutic Advances of RNAs in Precision Medicine of Gastrointestinal Tumors

**DOI:** 10.3390/biomedicines13010047

**Published:** 2024-12-28

**Authors:** Runhan Liu, Jiaxin Zhou, Xiaochen Chen, Jie Zhang, Qunzhi Chen, Xiaoming Liu, Kunhou Yao

**Affiliations:** 1 Department of Gastrointestinal Surgery, Huaihe Hospital of Henan University, Kaifeng 475000, China; 2 School of Clinical Medicine, Henan University, Kaifeng 475004, China; 3 School of Biomedical Engineering, University of Technology Sydney, Sydney, NSW 2007, Australia; 4 School of Life Sciences, Henan University, Kaifeng 475004, China; 5 School of Pharmacy, Henan University, Kaifeng 475004, China

**Keywords:** gastrointestinal tumors, RNA, diagnosis, drug resistance, precision medicine

## Abstract

Gastrointestinal tumors present a significant challenge for precision medicine due to their complexity, necessitating the development of more specific diagnostic tools and therapeutic agents. Recent advances have positioned coding and non-coding RNAs as emerging biomarkers for these malignancies, detectable by liquid biopsies, and as innovative therapeutic agents. Many RNA-based therapeutics, such as small interfering RNA (siRNA) and antisense oligonucleotides (ASO), have entered clinical trials or are available on the market. This review provides a narrative examination of the diagnostic and therapeutic potential of RNA in gastrointestinal cancers, with an emphasis on its application in precision medicine. This review discusses the current challenges, such as drug resistance and tumor metastasis, and highlights how RNA molecules can be leveraged for targeted detection and treatment. Additionally, this review categorizes specific diagnostic biomarkers and RNA therapeutic targets based on tissue type, offering a comprehensive analysis of their role in advancing precision medicine for gastrointestinal tumors.

## 1. Introduction

Gastrointestinal tumors, which develop within the digestive system, are varied and often highly fatal, encompassing types such as colorectal and gastric cancer [1]. Gastrointestinal tumors present major challenges for clinical diagnosis and treatment because of their biological complexity and heterogeneity. This complexity is evident not only across different tumor types but also within the same tumor, showing variation in cell differentiation levels, genetic and epigenetic features, and diversity within the tumor microenvironment [2]. Improving chemotherapy efficacy is crucial for enhancing cancer treatment outcomes [3]. However, the heterogeneity of gastrointestinal tumors often reduces the effectiveness of conventional therapies, which may not achieve the desired therapeutic results. This variability leads to significant differences in clinical outcomes and increases the risk of treatment failure and recurrence. To address these issues, precision medicine has emerged as a promising approach. The essence of this strategy is to develop personalized treatment plans based on comprehensive information, including the specific molecular features of a patient’s tumor, genetic mutations, epigenetic alterations, and individual metabolic profiles [4]. Precision medicine focuses not only on enhancing the accuracy of tumor diagnosis but also on optimizing therapeutic strategies. This is achieved by selectively targeting molecular markers and pathways specific to tumors, aiming to maximize therapeutic efficacy while minimizing harm to healthy tissues. As a result, precision medicine is increasingly acknowledged as a crucial approach for improving both the diagnostic precision and treatment outcomes for gastrointestinal tumors.

RNA-based diagnostics and therapeutics are transforming the management of Gastrointestinal tumors by leveraging RNA molecules such as microRNAs (miRNAs), small interfering RNAs (siRNAs), long non-coding RNAs (lncRNAs), aptamers, antisense oligonucleotides (ASOs), and messenger RNAs (mRNAs) for precise disease detection and targeted treatment. Non-coding RNAs serve as critical biomarkers, aiding in early diagnosis and prognosis through their presence in body fluids [5]. Treatment strategies include RNA interference (RNAi) to silence oncogenes [6], microRNA modulation to restore normal gene expression [7], ASOs to modulate RNA function [8], and mRNA vaccines designed to elicit immune responses against tumor-specific antigens [9]. Although challenges remain, such as improving delivery efficiency and ensuring RNA stability, these RNA-based approaches are paving the way for more effective and personalized treatments for gastrointestinal cancers.

This review aims to provide a comprehensive overview of the progress made in RNA-based diagnostics and therapeutics for gastrointestinal tumors. We discuss the mechanisms underlying RNA’s diagnostic and therapeutic potential, highlight its applications in overcoming drug resistance and metastasis, and explore emerging RNA targets for clinical intervention. By shedding light on the current advancements and challenges, this article seeks to underscore RNA’s transformative potential in the fight against Gastrointestinal cancers and guide future research toward effective and personalized treatment strategies.

## 2. Mechanistic Basis of RNA in Cancer Diagnosis

**Figure 1:**
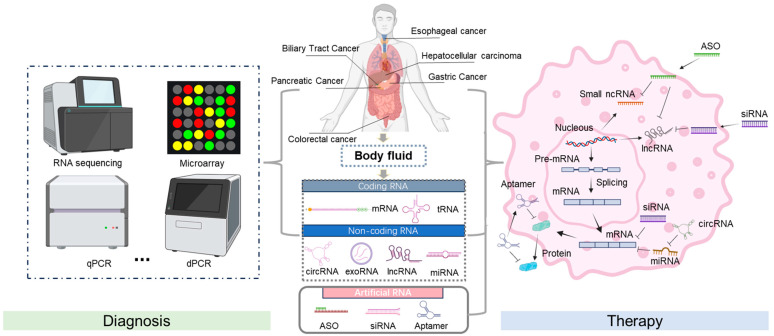
Schematic of RNA diagnosis and therapy. The screening of RNA biomarkers and RNA-based drugs for gastrointestinal cancers can focus on different stages of gene expression. This involves extracting RNA from samples and determining the disease status using specific techniques, such as RNA sequencing, microarray analysis, qPCR, dPCR, and subsequent data analysis. After entering the cell, it binds to the target RNA, promotes mRNA degradation or prevents its translation, thereby inhibiting the expression of harmful genes and achieving a therapeutic effect. Created using BioRender.com. Accessed in September 2024.

In cancer, RNA expression patterns are frequently altered, reflecting the genetic and epigenetic changes that contribute to tumorigenesis [10]. For gastrointestinal tumors, RNA can be isolated from cancer cells or various body fluids, such as blood, offering a minimally invasive diagnostic method. Distinct RNA signatures, characterized by upregulated or downregulated RNA species, can provide information about the presence of cancer, its type, and even the stage of the disease. Advanced technologies like RNA sequencing (RNA-Seq) facilitate comprehensive transcriptome analysis, enabling the sensitive and specific detection of these RNA biomarkers (Figure 1). In addition, RNA detection methods can reveal the activation of oncogenic pathways and the inhibition of tumor suppressor pathways, providing key insights into the molecular mechanisms that drive cancer progression [11].

### 2.1. Mechanistic Basis of Coding RNA in Cancer Diagnosis

Messenger RNAs (mRNAs), the main type of coding RNAs, are crucial in molecular biology as they bridge DNA and protein production. In cancer diagnosis, mRNAs and their derivatives, like fusion transcripts, are key markers. Abnormal expression levels of certain mRNAs in cancer indicate gene dysregulation associated with cell growth, apoptosis, and other vital pathways [12]. The analysis of mRNA expression profiles through techniques such as RNA-Seq enables the identification of unique mRNA signatures associated with different types of cancer. Fusion transcripts arise from chromosomal rearrangements that connect two previously independent genes, resulting in a novel mRNA that can promote oncogenesis. These fusion events are often specific to particular cancer types, making them significant diagnostic markers. For instance, the BCR-ABL fusion transcript is a defining marker for chronic myeloid leukemia (CML) [13], while EML4-ALK fusions are found in a subset of non-small-cell lung cancers [14]. Additionally, fusion transcripts such as FGFR2-GAB2 and NPC1-MELK have shown potential as diagnostic markers and therapeutic targets in esophageal adenocarcinoma [15]. NPM1-PTMA and HIST1H2BO-YBX1 fusion transcripts have a significant impact on the overall survival of patients with laterally spreading tumors of CRC and can be used as prognostic and diagnostic biomarkers [16].

In addition to mRNA, transfer RNA (tRNA) plays a vital role in diagnosing a variety of diseases. tRNAs are fundamental components of the cellular machinery that translate genetic information into proteins. Recent studies have shown that tRNAs and tRNA-derived fragments (tRFs) exhibit specific expression patterns across different cancer types, making them valuable biomarkers for disease detection and monitoring [17]. The diagnostic capabilities of tRNAs and tRFs stem from their stable presence in body fluids and their unique expression profiles associated with various diseases [18], reflecting changes in tumor metabolism, proliferation, and stress responses.

### 2.2. Mechanistic Basis of Non-Coding RNA in Cancer Diagnosis

ncRNAs, unlike coding RNAs, do not encode proteins but instead regulate gene expression at multiple levels [19]. The primary classes of ncRNAs relevant to cancer diagnosis include miRNAs, lncRNAs, circRNAs, exosome RNAs (exoRNAs) and ASOs.

miRNAs are small RNA molecules of approximately 22 nucleotides in length that control gene expression by binding to mRNA, thereby inhibiting its translation [20]. The dysregulation of certain miRNAs is associated with the onset and development of various types of cancer [21]. Dysregulation of miRNAs has been shown to promote cell cycle progression by downregulating the expression of CDK inhibitors in gastric carcinogenesis, and to regulate apoptosis by altering the expression of Bcl-2 family members in gastric cancer [22]. The dysregulation of miR-155 is closely related to *Helicobacter pylori*-related gastric disease, inflammatory bowel disease, and colorectal cancer, and is also involved in the molecular changes in important targets and signaling pathways [23]. lncRNAs, which are over 200 nucleotides in length, have various functions in gene regulation, including chromatin modification, transcriptional interference, and mRNA splicing [24], lncRNAs can act as molecular scaffolds, decoys, guides, or enhancers, affecting gene expression in a context-dependent manner. In cancer, lncRNAs are often dysregulated, contributing to tumorigenesis and metastasis [25]. circRNAs are a unique class of ncRNAs formed by the back-splicing of pre-mRNAs, resulting in covalently closed loop structures [26]. In cancer, circular RNAs (circRNAs) can affect critical signaling pathways and are often expressed differently than in normal tissues [26]. The stability and abundance of circular RNA in body fluids underpins their potential as promising biomarkers for cancer detection and monitoring of therapeutic responses. Exosomes are extracellular vesicles ranging from 40 to 160 nanometers in size that are secreted by various cell types, including cancer cells [27]. Consequently, analyzing exoRNAs can yield critical information for the early detection of cancer and other diseases. Antisense oligonucleotides (ASOs) possess a unique ability to bind to complementary RNA sequences with high affinity, allowing for precise targeting of specific mRNA molecules and facilitating the identification of gene expression patterns indicative of disease [28]. Moreover, ASOs can be designed to distinguish between similar RNA sequences, including those featuring single-nucleotide polymorphisms, thereby enhancing their relevance in personalized medicine [29]. The diagnostic applications of ASOs encompass a range of diseases, including cancers, neurodegenerative disorders, and viral infections [30]. RNA diagnostics plays a vital role in early disease detection, treatment decisions, and long-term management.

## 3. Therapeutic Applications of RNA

Compared with traditional therapies, RNA therapy can more accurately target the molecular mechanisms in pathological and physiological processes, thereby achieving personalized treatment of diseases. In recent years, RNA technology has achieved remarkable results in the fields of infectious diseases, cancer, genetic diseases, neurodegenerative diseases, metabolic disorders, etc., especially the clinical application of mRNA vaccines, siRNA, aptamers, ASOs and other technologies, which has brought new hope to human health (Table 1).

### 3.1. Therapeutic Applications of Coding RNA

The core concept of mRNA therapeutics involves delivering synthetic mRNA into cells, where it serves as a template for protein synthesis. This enables the production of therapeutic proteins to address genetic defects, fight infection or stimulate an immune response. Key therapeutic applications of mRNA include vaccines, cancer immunotherapy, protein replacement therapy, regenerative medicine, and antiviral and antibacterial therapies [31]. miRNAs can function either as oncogenes due to their success in combating infectious diseases, most notably COVID-19 [32]. For example, Pfizer-BioNTech (BNT162b2) and Moderna (mRNA-1273) have both received formal commercial approval from the FDA [32]. mRNA-4650 vaccine improves adoptive T cell therapy of neoantigen-specific cells in patients with metastatic gastrointestinal cancer without severe toxic side effects [33]. In addition to the typical function of tRNA in protein synthesis, tRNA and its derivatives have emerged as promising tools for therapeutic applications. Suppressor tRNAs are engineered to recognize and read through premature stop codons, enabling the translation machinery to generate full-length, functional proteins even in the presence of mutations. For example, ACE tRNA successfully promoted the readthrough of stop codons in cystic fibrosis airway epithelial cells in vitro and has shown promise in treating conditions such as cystic fibrosis and Duchenne muscular dystrophy [34]. The natural stability and cellular uptake mechanisms of tRNA can be harnessed to enhance the bioavailability and effectiveness of these conjugated therapeutics [35]. 

### 3.2. Therapeutic Applications of Non-Coding RNA

ncRNAs are able to regulate gene expression and influence cellular processes without coding for proteins. These molecules play an important role in the development and progression of diseases such as genetic disorders, cancer, and cardiovascular disease [19]. 

miRNAs can function either as oncogenes or tumor suppressors, which makes them significant targets for cancer therapies. Therapeutic approaches often involve employing miRNA mimics or inhibitors to restore their normal functions. MRX34 is the first miRNA mimic to enter phase 1 studies. MRX34 can restore the lost inhibitory function of endogenous miR-34 and is involved in regulating 24 known oncogenes, including antiapoptosis, chemoresistance, and cancer cell self-renewal [36]. When the expression of tumor suppressor miRNAs, such as members of the Let-7 and miR-34 families, is reduced, tumor invasion and metastasis are enhanced. Synthetic miRNA mimics can restore tumor suppressor miRNA function in tumor cells [37]. Similarly, lncRNAs present promising therapeutic opportunities in cancer treatment due to their roles in regulating gene expression and cellular processes. Strategies for targeting lncRNAs include utilizing RNA interference or antisense oligonucleotides to inhibit oncogenic lncRNAs, gene therapy to restore tumor suppressor lncRNAs, and employing small molecules or peptides to disrupt lncRNA-protein interactions [38]. Nuclear-specific RNA interference (RNAi) nanoplatforms provide a precise method for regulating nuclear lncRNA functions, offering an effective strategy for cancer treatment [39]. Additionally, engineered lncRNAs can serve as targeted drug delivery systems, enhancing the precision and efficacy of therapeutic interventions [40]. circRNAs also play dual roles as oncogenes or tumor suppressors, and modulating their expression can impact cancer progression [41]. Therapeutic strategies for circRNAs include using mimics or inhibitors to restore their normal functions. For example, circRNAs can be designed to act as miRNA sponges, capturing oncogenic miRNAs to prevent them from suppressing tumor suppressor genes [42,43]. hsa_circ_0005785, which is overexpressed in Acute myeloid leukemia, may act as an oncogene by sponging miR-181 family miRNAs and driving tumorigenesis [44]. Cdr1as can bind to miR-7 and act as a miRNA sponge. In pancreatic islet cells, Cdr1as regulates insulin transcription and secretion through miR-7 and its targets [45]. Inhibiting circRNAs that facilitate cancer cell proliferation can effectively reduce tumor growth. ExoRNAs serve dual purposes as both biomarkers for cancer diagnosis and therapeutic agents. By examining the RNA content of exosomes obtained from cancer patients, clinicians can identify specific RNA signatures linked to various cancer types, stages, and treatment responses. Therapeutically, exosomes can be designed to deliver RNA molecules targeting oncogenes or to restore the function of tumor suppressor genes. For example, exosomes infused with miRNAs or siRNAs that target genes promoting cancer can be utilized to hinder tumor growth and metastasis [46]. For example, therapeutic si*S100A4* loaded into exosomes can be delivered to the lungs and mediate cancer treatment in mice with lung metastases after surgery [47]. siRNA targeting *SIRT6* loaded into exosomes and modified with E3 aptamers showed a strong targeted therapeutic effect in subcutaneous prostate cancer mice, reducing orthotopic tumor growth and liver metastasis burden [48]. Adipose tissue-derived mesenchymal stem cells (AMSCs) transfected with miR-122 can effectively package miR-122 into secreted exosomes, thereby mediating miR-122 communication between AMSCs and HCC cells, thereby sensitizing cancer cells to chemotherapeutic drugs by altering the expression of miR-122 target genes in HCC cells [49]. Moreover, RNA interference can degrade SARS-CoV-2 spike protein RNA, presenting a potential avenue for developing treatments for COVID-19 [50]. ASOs affect the processing, translation, and stability of specific target RNAs. In cancer therapy, ASOs can modulate the expression of oncogenes and tumor suppressor genes. For example, ASOs targeting the antiapoptotic gene *BCL-2* can induce apoptosis in cancer cells, thereby improving chemotherapy outcomes and reducing drug resistance [51].

## 4. RNA-Based Strategies for Drug Resistance and Tumor Metastasis

### 4.1. RNA Therapeutic Strategies to Overcome Tumor Drug Resistance

Drug resistance is a major obstacle to cancer treatment and can be caused by mechanisms such as enhanced drug efflux, altered drug targets, activation of survival pathways, and changes in the tumor microenvironment [52]. 

miRNAs can modulate these resistance mechanisms by simultaneously targeting multiple genes and pathways, thus providing a promising strategy to counteract drug resistance in cancer therapy. They can also influence the tumor microenvironment, immune response, and cellular communication, further aiding in the reversal of resistance [53]. Certain miRNAs, such as miR-21, promote drug resistance by downregulating tumor suppressor genes. Inhibitors like antagomiRs can restore the expression of these tumor suppressor genes, making cancer cells more sensitive to chemotherapy [54]. Additionally, miRNA mimics can be used to elevate their levels, thereby inhibiting pathways that contribute to resistance; for instance, miR-34 mimics can suppress the expression of genes associated with chemoresistance [55]. siRNAs can be specifically designed to target and degrade mRNAs encoding proteins that facilitate drug resistance, effectively silencing the genes responsible. Drug efflux pumps such as P-glycoprotein (encoded by the *MDR1* gene) can expel chemotherapy drugs from cancer cells, thereby reducing the effectiveness of treatment. By targeting *MDR1* with siRNAs, the expression of these pumps can be reduced, leading to increased intracellular drug concentration and enhanced chemotherapy effectiveness [56], which can be specifically designed to target and degrade mRNAs encoding proteins that facilitate drug resistance, effectively silencing the genes responsible. lncRNAs also play critical roles in regulating gene expression and have been associated with drug resistance; for example, HOTAIR is linked to chemoresistance. Inhibiting HOTAIR with antisense oligonucleotides (ASOs) or siRNAs can disrupt its function, thereby increasing the sensitivity of cancer cells to chemotherapeutic agents [57].

Combining RNA-based therapies with traditional cancer treatments can create a synergistic effect and help overcome drug resistance. By combining siRNA or miRNA inhibitors with chemotherapy, RNA therapy can specifically target resistance mechanisms, while chemotherapy acts directly on cancer cells [58]. RNA therapeutics can also be combined with targeted therapies to prevent the emergence of resistant cell populations. For example, combining miRNA mimics that suppress resistance pathways with kinase inhibitors can enhance the durability of the response [59]. Lipid nanoparticles (LNPs) can encapsulate RNA molecules, protecting them from degradation and aiding in their delivery to target cells [60,61]. They are widely used in delivering siRNAs and miRNA mimics. Exosomes can be engineered to carry RNA therapeutics. They offer a biocompatible and targeted delivery method, capable of crossing biological barriers and delivering cargo to specific cells [62].

### 4.2. The Role and Application of RNA in Inhibiting Tumor Metastasis

The spread of cancer cells from the primary tumor to distant organs, known as tumor metastasis, is a leading cause of cancer-related deaths [63]. Specific miRNAs can act as metastasis suppressors or promoters. For example, antagomiRs targeting miR-210 can restore the expression of tumor suppressor genes and reduce metastatic potential [64]. miR-200 family members and miR-34 function as metastasis suppressors by inhibiting epithelial-mesenchymal transition (EMT), a key process in metastasis [65]. Delivering miRNA mimics to restore the levels of these miRNAs can help prevent cancer cell invasion and dissemination. siRNA can induce the degradation of specific mRNA targets, effectively silencing gene expression. siRNAs can be designed to target genes involved in metastasis. siRNAs targeting key EMT regulators, can inhibit EMT and reduce metastatic potential [66]. Matrix metalloproteinases (MMPs) break down the extracellular matrix, promoting cancer cell invasion. siRNAs targeting specific MMPs, like MMP-2 and MMP-9, can reduce their expression, thereby limiting the invasive capacity of cancer cells in surrounding tissues [67]. lncRNAs can influence metastasis either by promoting or suppressing it. lncRNAs can modulate key signaling pathways involved in metastasis, such as the Wnt/β-catenin, PI3K/AKT, and NF-κB pathways [68]. Oncogenic lncRNAs can stimulate pathways that drive metastasis, while tumor suppressor lncRNAs work to inhibit these pathways, preventing cancer from spreading. Restoring tumor suppressor lncRNA expression can, therefore, help block metastasis. Gene therapy techniques can be employed to deliver functional copies of lncRNAs like growth arrest-specific 5 (GAS5) and MEG3 into cancer cells, thereby restoring their inhibitory effects on metastasis-related pathways [69]. Synthetic mimics of tumor suppressor lncRNAs can be introduced into cells to replicate their function. These mimics can help suppress metastatic behaviors by modulating gene expression and signaling pathways.

## 5. RNA Diagnosis and Therapeutic of Gastrointestinal Tumors

Identifying RNA diagnostic and therapeutic targets is critical in improving the management of gastrointestinal tumors. Targeting these RNA molecules can facilitate the development of more precise therapeutic strategies. Various RNA types such as miRNA and circRNA. have shown potential as diagnostic and therapeutic targets. The following sections will delve into specific RNA targets associated with gastrointestinal tumors and illustrate their potential in improving diagnostic accuracy and therapeutic efficacy. 

### 5.1. RNA Diagnosis and Therapeutic in Colorectal Cancer

Colorectal cancer (CRC) is the second leading cause of cancer death in the United States. Approximately 153,000 new cases are diagnosed each year, with approximately 53,000 fatalities. The median age of diagnosis is 66, and the lifetime risk of developing CRC is 4.3%. CRC typically originates from a benign adenomatous polyp, which may progress to an advanced adenoma and eventually transform into invasive cancer [70]. The 5-year survival rate for CRC is approximately 90% when detected early. However, if the cancer spreads beyond the colon or rectum, the survival rate declines sharply. Thus, early detection is essential for improving CRC patient outcomes. Currently, the main screening methods for CRC include imaging techniques and stool-based tests [71]. However, colonoscopy and other techniques cannot be routinely performed in all patients due to factors such as sensitivity, specificity, time, cost, and patient eligibility [72]. Oxaliplatin is commonly used as a first-line chemotherapy treatment for colorectal cancer. However, changes in higher-order chromatin conformation cause colorectal cancer cells to acquire oxaliplatin resistance [73]. RNA-based diagnostics and therapeutics present a promising advancement in CRC management.

#### 5.1.1. RNA Diagnostic Biomarkers in CRC

**Figure 2:**
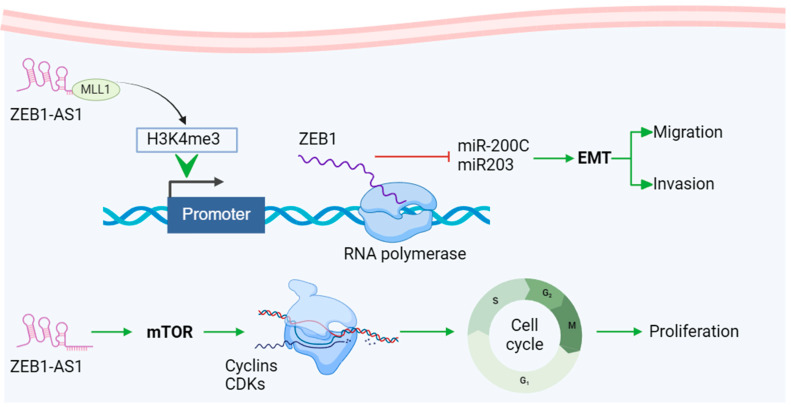
Mechanisms of representative lncRNA biomarker ZEB-AS1 in CRC. lncRNA ZEB1-AS1 recruits H3K4 methyltransferase MLL1 to the *ZEB1* promoter and induces H3K4me3 modification in this region, promoting *ZEB1* transcription. ZEB1 promotes EMT (epithelial-mesenchymal transition) and accelerates tumor migration and invasion by regulating downstream molecules such as miR200c and miR203. In addition, lncRNA ZEB1-AS1 can activate the mTOR pathway, promote Cyclin D1 expression, and promote G1 cells to enter the S phase. The upregulation of Cyclin D1 enhances its binding activity with CDK4/CDK6, further accelerating the cell cycle and promoting tumor cell proliferation. Created using BioRender.com, accessed on 1 December 2024. Adapted with permission from Refs. [77,78].

miRNAs such as miR-21, miR-92a, and the miR-20a family are important for non-invasive early detection and monitoring, as their expression changes are linked to tumor progression and prognosis. Additionally, certain circRNAs show promise as prognostic biomarkers for CRC. For example, the significant upregulation of circHIPK3 in CRC tissues and cell lines was closely associated with tumor metastasis and advanced clinical stages, indicating its potential as a predictive biomarker for CRC [74]. In CRC tissues, circ_0003906, circCDC66, and other circRNAs have been identified and clinically validated through qRT-PCR and RNA-Seq [75]. The molecular pathway of lncRNA ZEB1-AS1 is a typical representative of the mechanism of lncRNA involved in tumor regulation. As a representative lncRNA, its unique target *ZEB1* can combine with EMT-related pathways and play a particularly important role in cancer metastasis, invasion, and cell cycle regulation. This has become one of the hot spots in tumor marker research and provides a new direction for anticancer treatment targets (Figure 2) [76]. As diagnostic markers for CRC, these RNAs can accurately detect cancer at an early stage and provide personalized treatment information, thereby improving patient prognosis.

#### 5.1.2. Therapeutic RNA Targets in CRC

Targeting specific RNAs offers an effective therapeutic approach to inhibit cancer-promoting pathways. For example, miR-185 can reduce the metastatic ability of CRC cells by inhibiting stromal interaction molecule 1 (STIM1) [79]. siRNAs have multiple applications, including suppressing CRC cell growth, inducing apoptosis, overcoming drug resistance, and preventing metastasis. A study shows that siRNA targeting *KRAS* inhibits the proliferation of CRC cells and slows tumor growth in mouse models [80]. Additionally, silencing *MALAT1* with siRNA reduced invasion, metastasis, and cell proliferation while increasing apoptosis in CRC cells [81]. ASOs can also downregulate oncogenes; for example, targeting circLONP2 with ASOs significantly reduced CRC metastasis to other organs by decreasing nodule size and number [82]. Growth factors like epidermal growth factor receptor (EGFR) are crucial for CRC cell proliferation, and using EGFR ASO encapsulated in PAMAM nanoparticles has been shown to reduce the proliferation of the human colon cancer cell line HT29 [83]. These RNA-based approaches enable precise targeting of cancer cells, enhancing the efficacy of CRC treatment while minimizing side effects.

### 5.2. RNA Diagnosis and Therapeutic in Gastric Cancer

Gastric cancer (GC) is often diagnosed at an advanced stage, resulting in high mortality. GC is the third leading cause of cancer death and the fifth most common malignancy worldwide, with more than 1 million new cases each year [84]. The causes of gastric adenocarcinoma are diverse, and the main risk factors include dietary factors, such as high salt intake, smoking, and Helicobacter pylori infection. [84]. Surgical resection remains the most effective treatment for GC, while chemotherapy and radiotherapy serve as crucial adjuvant therapies. However, with an aging population, the number of elderly patients is rising, leading to more complex treatment protocols and a higher risk of complications [85]. Therefore, the prevention and early diagnosis of GC are very important. However, there is currently a lack of convenient and effective early screening methods.

#### 5.2.1. RNA Diagnostic Biomarkers in GC

miRNAs exhibit abnormal expression not only in gastric cancer tissues but also in various body fluids, positioning them as potential biomarkers for the disease. Furthermore, alterations in miRNA expression within exosomes further strengthen their utility as biomarkers. For instance, studies have shown that the expression level of miR-221 in the peripheral blood exosomes of gastric cancer patients is significantly higher than that in healthy individuals [86]. Research has shown that the levels of hsa_circ_0000181 are significantly lower in the tissues and plasma of GC patients compared to non-tumor tissues and healthy plasma [87]. The decrease in hsa_circ_0003159 in patients’ plasma was closely correlated with carcinoembryonic antigen levels, indicating that it has the potential to become a diagnostic biomarker for GC [88]. 

#### 5.2.2. Therapeutic RNA Targets in GC

Chemotherapy is commonly used to extend survival in patients with advanced disease. However, the effectiveness of chemotherapy is often limited by multidrug resistance. Multidrug resistance-associated protein (MRP-1), alongside P-glycoprotein, acts as a transporter that expels cytostatic drugs from cells. Transfecting drug-resistant cells with miR-326 has been shown to lower MRP-1 expression, increasing the cells’ sensitivity to VP-16 and doxorubicin [89]. Therapeutically, ASOs and siRNA targeting oncogenes like *HER2*, *KRAS*, and *VEGF*, as well as lncRNAs such as HOTAIR and MALAT1, and circRNAs like circPVT1 and circ_0000523, can inhibit cancer-promoting pathways and reduce tumor growth and metastasis. Wang et al. found that overexpression of circ_0027599 can inhibit the proliferation and metastasis of GC cells [90]. These RNA-based strategies enable precise targeting of cancer cells, offering improved treatment efficacy and reduced side effects, ultimately enhancing patient outcomes in GC management.

### 5.3. RNA Diagnosis and Therapeutic in Esophageal Cancer

Esophageal cancer (EC) is mainly divided into esophageal squamous cell carcinoma (ESCC) and esophageal adenocarcinoma (EAC). ESCC represents the majority of cases worldwide, particularly in East Asia and Africa, while EAC is more frequently seen in developed countries [91]. The disease is often asymptomatic in its early stages, leading to late diagnosis when the cancer has already progressed or metastasized. Traditional treatments include surgery, which is typically used for localized tumors, chemotherapy and radiation therapy for more advanced stages, and targeted therapies aimed at specific molecular pathways involved in tumor growth. Despite the availability of various treatment options, challenges like drug resistance, high recurrence rates, and severe side effects that affect the patient’s quality of life remain significant. In recent years, advancements in radiotherapy, chemotherapy, and surgical techniques have led to substantial progress in treating EC. However, early diagnosis is still uncommon and remains a critical issue to address.

#### 5.3.1. RNA Diagnostic Biomarkers in EC

The poor prognosis of EC patients is partly due to the lack of effective diagnostic markers and therapeutic targets. Therefore, identifying biomarkers or therapeutic targets is crucial to improve clinical outcomes for EC. Due to their expression patterns and characteristics, circRNAs are promising as ideal biomarkers. QRT-PCR validation and receiver operating characteristic (ROC) curve analysis demonstrated that circ_0004771 has significant diagnostic potential, with its expression level strongly correlated with T grade (tumor invasion extent) and vascular invasion. This suggests that circ_0004771 could serve as a prognostic indicator [92]. The detection of miRNA and lncRNA can be used as biomarkers for early diagnosis and prognosis. Microarray analysis found that miR-574-3p, miR-1303, miR-1909, miR-204, and miR-886-3p were differentially expressed in patients with postoperative tumor recurrence and those without recurrence [93]. lncRNA CCAT2 is upregulated in EC tissues, and its high expression is strongly linked to poor overall survival in these patients. Those with elevated CCAT2 levels face a higher risk of cancer-related mortality, indicating that CCAT2 could serve as both a predictive biomarker and a therapeutic target for EC [94]. 

#### 5.3.2. Therapeutic RNA Targets in EC

miR-100 functions as a tumor suppressor in EC and could be a potential treatment option [95]. The miR-124/CDK4 axis plays a crucial role in regulating the radiosensitivity of human EC cells, suggesting that targeting CDK4 may enhance radiotherapy efficacy [96]. Additionally, targeting β-elemene-mediated lncRNA CDKN2B-AS1 has been shown to reduce cell proliferation and promote apoptosis by decreasing hTERT levels [97]. Clinically, cisplatin, fluorouracil, and paclitaxel are used to treat ESCC. CircDOCK1 (hsa_circ_0007142), originating from DOCK1, is upregulated in cisplatin-resistant ESCC tissues and cells and contributes to resistance by increasing LIM and SH3 protein 1 (LASP1) through miR-494-3p sponging [98]. The study found that exosome-mediated has_circ_0000337 induced cisplatin (chemotherapeutic drug) resistance in ESCC cells through the miR-377-3p/JAK2 axis. At the same time, resistant ESCC exosomes carrying circPPFIA1 can promote the resistance, proliferation and metastasis of cisplatin-sensitive ESCC cells in vivo and in vitro [99]. These RNA molecular targets offer new insights and opportunities for treating EC and assessing prognosis. Exploring the mechanisms of RNA action in EC and identifying effective RNA-based therapeutic targets are crucial for clinical advancements.

### 5.4. RNA Diagnosis and Therapeutic in Pancreatic Cancer

Pancreatic cancer (PC) is the leading cause of cancer-related death, and its overall prognosis has long been poor. Pancreatic ductal adenocarcinoma (PDAC) is the most common type, with only approximately 11% of patients surviving more than 5 years after diagnosis, and remains a major challenge in oncology. Nevertheless, most patients experience relapse due to micrometastatic disease [100]. RNA has unique advantages as a diagnostic marker and therapeutic target. 

#### 5.4.1. RNA Diagnostic Biomarkers in PC

Multiple studies have identified circulating tumor-derived miRNAs as diagnostic biomarkers for PC. Wang et al. found that miR-21, miR-210, miR-155, and miR-196a were upregulated in the plasma of PDAC patients [101], suggesting their potential as biomarkers, as they are also elevated in PC tissues and cell lines. Similarly, miR-200a/b, miR-18a, miR-221, and miR-196a/b were expressed in cancer tissues and their levels were increased in serum/plasma [102]. Through screening of the Gene Expression Omnibus database, the expression level of circRNA hsa_circ_0013587 was associated with PC progression [103]. Lu Hong et al. found that hsa_circ_0006220 and hsa_circ_0001666 were highly expressed in plasma exosomes of PC patients compared with healthy controls [104]. LINC00675 (lncRNA) overexpression is strongly linked to lymph node metastasis, perineural invasion, and poor clinical outcomes in pancreatic cancer (PC) patients. It also serves as a potential diagnostic marker for predicting recurrence following radical surgical resection in PDAC patients [105]. RNA interference technologies such as siRNA and ASO can target oncogenes such as *KRAS* and *BCL-2* to inhibit tumor growth and progression. 

#### 5.4.2. Therapeutic RNA Targets in PC

Oncogenic miRNAs such as miR-21, miR-221, and miR-320a are known to cause drug resistance in PC cells. miR-21 specifically targets PTEN and PDCD4 through the PI3K/AKT/mTOR pathway to promote tumor growth. In addition, upregulation of PTEN and PDCD4 may reduce the effect of miR-21 on drug resistance in PC [106]. Recent studies have shown that circRNAs influence PC sensitivity to chemotherapy [107]. CircHIPK3 (hsa_circ_0000284) has been implicated in tumor development and chemoresistance across various cancers. In a recent study, Professor Zhu’s team discovered that circHIPK3 interacts with miR-330-5p, enhancing gemcitabine resistance and promoting cell proliferation and progression by upregulating RASSF1 expression in PC [107]. *ATG7* and *WIF-1* are key genes in the WNT pathway that have an important impact on radiosensitivity. Researchers Jiang et al. found that silencing HOTAIR leads to upregulation of ATG7 and WIF-1, thereby regulating autophagy. This discovery reveals the potential role of HOTAIR in the regulation of autophagy and radiosensitivity, and may provide new ideas for cancer treatment. As a result, the HOTAIR/ATG7/WIF-1 axis could serve as a potential therapeutic target to enhance the effectiveness of radiotherapy in PDAC [108]. The lncRNA GAS5 plays an important tumor suppressor role in PC. There is also evidence that GAS5 is involved in chemoresistance in PC cells, and GAS5 overexpression promotes the efficacy of chemotherapy in PC mouse models [69]. These RNA targets can not only provide accurate diagnostic information, but also improve treatment effects by regulating gene expression, representing the cutting-edge direction of PC research and treatment.

### 5.5. RNA Diagnosis and Therapeutic in Hepatocellular Carcinoma

Hepatocellular carcinoma (HCC) is the most common type of liver cancer, accounting for approximately 90% of all liver cancer cases [109]. HCC is often linked to chronic hepatitis virus infection, cirrhosis, and long-term alcohol consumption. Although there are many treatment methods such as surgical resection, local ablation, interventional therapy, and targeted therapy. [110] and immunotherapy, many patients are diagnosed at an advanced stage due to the absence of clear early symptoms. HCC represents a global health issue with rising rates of incidence and mortality. Despite the increasing availability of surgical and local therapies worldwide, approximately 50–60% of HCC patients eventually require systemic treatment [111]. The treatment effect is often unsatisfactory and the recurrence rate is high. 

#### 5.5.1. RNA Diagnostic Biomarkers in HCC

The overall survival rate of patients with liver cancer is low, largely due to the lack of effective biomarkers for accurate early diagnosis. Changes in miR-21, miR-122, and HULC levels can be used for early diagnosis and prognosis assessment of HCC. HULC is highly expressed in HCC, and its inhibition can reduce tumor growth and improve chemotherapy sensitivity. Since circRNAs have high stability and abundance in liver cancer tissues and body fluids, such as hsa_circ_0005075 and hsa_circ_0091579, they are ideal sources for developing new biomarkers for early diagnosis of liver cancer [112]. Studies have shown that lncRNAs in serum, small nucleolar RNA host genes, lncRNA uc007biz.1 (LRB1) and linc00152, which are significantly upregulated in liver cancer, are potential biomarkers for early hepatocellular carcinoma (HCC). In addition, combining the detection of these lncRNAs with serum AFP levels can provide the highest sensitivity and accuracy for early HCC diagnosis [113]. 

#### 5.5.2. Therapeutic RNA Targets in HCC

Long non-coding RNA plays a crucial role in the development, progression, and drug resistance of HCC. MKLN1-AS levels are notably elevated in HCC tissues, and this upregulation is clinically linked to vascular invasion, disease-free survival, and reduced overall survival in HCC patients. Functionally, silencing MKLN1-AS significantly suppressed HCC cell growth and metastasis both in vitro and in vivo. Research by Xijun Chen et al. indicates that MKLN1-AS could be a potential prognostic marker and therapeutic target for HCC treatment [114]. Through RNA interference technology, such as siRNA and ASO, targeting genes such as *VEGF* and *TGF-β* can inhibit tumor angiogenesis and cell proliferation, and improve treatment effects [115]. Circ_0051443 levels are significantly lower in the plasma exosomes and tissues of HCC patients compared to healthy controls, with the majority of circ_0051443 packaged into exosomes. ROC analysis indicates that exosomal circ_0051443 can effectively distinguish HCC patients from controls, making it a potential predictive biomarker and therapeutic target for HCC [116]. Rong Li et al. discovered that the novel circular RNA circ_102,166 is downregulated in HCC and that its expression level is closely associated with various clinicopathological features and the clinical prognosis of HCC patients. The overexpression of circ_102,166 was shown to significantly suppress the proliferation, invasion, migration, and tumorigenicity of HCC cells [117]. The diversity and specificity of RNA molecules give them great potential in the diagnosis and treatment of HCC, providing a new direction for improving diagnostic accuracy and treatment effects.

### 5.6. RNA Diagnosis and Therapeutic in Biliary Tract Cancer

Biliary tract cancer (BTC), which includes intrahepatic and extrahepatic bile duct cancer, gallbladder cancer, and ampullary cancer, is a heterogeneous group of malignancies that are usually diagnosed at an advanced stage when radical surgery is not possible, resulting in a poor prognosis [118]. BTC accounts for approximately 3% of gastrointestinal cancer cases in the United States and is the second most common primary liver cancer [119]. Treatment options include surgical resection, chemotherapy, radiotherapy, targeted therapy, and immunotherapy. However, due to the lack of obvious early symptoms, BTC is often diagnosed at an advanced stage. Coupled with the complex location of the tumor and resistance to treatment, the therapeutic effect is severely limited. 

#### 5.6.1. RNA Diagnostic Biomarkers in BTC

RNA shows unique advantages in the diagnosis and treatment of BTC. A study by Yueting Han et al. found that five types of miRNAs were significantly upregulated in the serum of patients with cholangiocarcinoma. miR-221, along with miR-10a, miR-21, miR-135b, and miR-214, can serve as early diagnostic markers for cholangiocarcinoma and other biliary tract diseases [120]. The study by X.-M. JIANG et al. found that upregulated lncRNA CCAT1, which is associated with malignant and aggressive behavior, could be used as a novel prognostic biomarker and a potential therapeutic target for cholangiocarcinoma [121]. The expression of lncR-AFAP1-AS1 in tumor tissues was significantly higher than that in para-cancerous tissues. And after reducing the expression of AFAP1-AS1, the tumor volume was significantly reduced and the growth was inhibited. It can be seen that NEAT1 and AFAP-1 are significantly related to tumor growth, differentiation and metastasis. Therefore, both can be used as potential early diagnostic markers and therapeutic targets for cholangiocarcinoma [122]. Jiang et al. discovered that circRNA-Cdr1as expression was significantly elevated in tumor tissue compared to the normal tissue adjacent to it. Multivariate analysis indicated that circRNA-Cdr1as could serve as an independent biomarker for predicting the prognosis of cholangiocarcinoma [123]. siRNA and ASO technologies can target genes like *KRAS* and *EGFR*, effectively inhibiting tumor cell proliferation and metastasis, and significantly enhancing treatment outcomes. Additionally, the abnormal expression of lncRNAs such as H19 and MALAT1 in BTC is strongly associated with tumor progression, suggesting that targeting these lncRNAs could offer new therapeutic strategies. The high specificity and regulatory capabilities of RNA molecules demonstrate significant potential for precise diagnosis and personalized BTC treatment, paving the way for new research and therapeutic directions.

#### 5.6.2. Therapeutic RNA Targets in BTC

Although chemotherapy is the main treatment for cholangiocarcinoma besides surgery, existing basic experiments have found that miRNA can regulate the sensitivity of cholangiocarcinoma tissue to chemotherapy drugs. Peng et al. found that the expression of miR-200b and miR-200c in the miR-200 family was significantly downregulated in cholangiocarcinoma tissue, and the sensitivity of tumor cells to fluorouracil was greatly improved after upregulating these two miRNAs in subsequent experiments [124]. Other studies have found that after upregulating the expression levels of miR-205, miR-221, and miR-29B, the sensitivity of tumor cells to gemcitabine is also improved [125]. Xu et al. found that hsa_circ_0001649 overexpression inhibited cell proliferation, migration and invasion, and hsa_circ_0001649 could induce tumor suppression by inducing apoptosis of biliary cancer cells KMBC and Huh-28 [126]. *KRAS* mutations are frequently observed in many cancers, including BTC, and are linked to tumor growth and therapy resistance. RNA-based therapies, such as siRNA or ASOs that target *KRAS*, can effectively reduce its expression, which may lead to decreased tumor growth and improved treatment responses [127]. This type of RNA therapy provides a highly focused, personalized, and potentially curative strategy for treating BTC. Its capacity to target the specific genetic and molecular drivers of BTC represents a significant advancement in combating this challenging and often lethal disease.

## 6. Prospect

The incorporation of RNA-based technologies into cancer diagnostics and therapeutics has led to significant progress, especially in the early detection and treatment of gastrointestinal tumors. RNA molecules provide high specificity and sensitivity, facilitating non-invasive tests for the early diagnosis of cancers. Moreover, RNA-based therapeutics offer personalized treatment options by specifically targeting oncogenes or tumor suppressors, effectively decreasing tumor growth and metastasis while minimizing off-target effects. However, several challenges remain in the efficient delivery of RNA molecules to targeted cells and tissues, as RNA is susceptible to degradation by nucleases present in the bloodstream [128]. The production of RNA therapies involves complex resource-intensive processes, including enzyme synthesis, purification, and advanced formulation, making large-scale production difficult and expensive. High production costs caused by expensive raw materials and delivery systems such as lipid nanoparticles (LNPs) further limit accessibility. Efficient and targeted delivery poses additional challenges, especially for tissues with complex barriers such as the brain or lungs, as the delivery vehicle must ensure protection from degradation, effective absorption, and intracellular release without causing toxicity [129]. Precision therapies utilizing RNA technologies also struggle with ensuring targeted delivery and avoiding unintended gene silencing or activation [130]. Addressing these obstacles is essential for fully harnessing the potential of RNA in delivering effective, personalized, and accessible cancer care.

### 6.1. Improve Clinical Sensitivity and Treatment Effectiveness

Enhancing the clinical sensitivity and therapeutic effectiveness of RNA diagnostics and treatments is crucial in the ongoing fight against cancer and other diseases. By improving clinical sensitivity, it becomes possible to detect illnesses in their earliest stages, often before symptoms manifest. Early diagnosis significantly boosts the chances of successful treatment and contributes to higher long-term survival rates. Advancements in RNA diagnostics can yield more accurate insights into the presence and progression of diseases, thereby minimizing false positives and false negatives [131]. This level of precision guarantees that patients receive timely and appropriate care based on accurate diagnoses. Additionally, by thoroughly profiling the genetic and molecular characteristics of an individual’s condition, RNA diagnostics can inform the creation of personalized treatment strategies [132]. This tailored approach ensures that therapies are designed to target the specific attributes of a patient’s disease, enhancing effectiveness and minimizing side effects. Personalized treatments guided by precise RNA diagnostics can also improve prognostic evaluations, enabling clinicians to more accurately predict disease outcomes and adjust treatment plans as needed.

### 6.2. Achieving Precision Medicine

The goal of precision medicine is to customize medical treatments based on each patient’s unique characteristics, including their genetic profile, lifestyle, and environment. RNA diagnostics and therapies are at the forefront of modern medical approaches, providing significant advancements in the personalization and effectiveness of healthcare. Imaging technology plays a crucial role in RNA therapy by enabling real-time monitoring of RNA delivery accuracy, ensuring that therapeutic agents reach their intended targets effectively. This technology also allows for the assessment of therapeutic effects and tumor responses, facilitating the optimization of treatment plans and enhancing overall efficacy. NIR-II imaging, as a non-invasive visual inspection technique, holds great promise for understanding tumor heterogeneity and progression differences [133,134]. Fe_3_O_4_ is particularly suitable for magnetic resonance imaging due to its superior magnetic response characteristics [135]. It is crucial to effectively deliver RNA molecules to target tissues and cells. An ideal delivery system can safely transport RNA to target cells while protecting RNA from degradation enzymes and reducing side effects caused by non-specific targeting. Such delivery systems include lipid nanoparticles, polymer nanomaterials, liposomes, and inorganic nanoparticles (such as gold nanoparticles, quantum dots). These carriers can be used to protect RNA, enhance targeting, and transport it into cells. For example, LNPs can prevent nuclease degradation and promote cellular uptake by encapsulating RNA molecules in lipid carriers [136]; exosomes use the stability of natural exosome vesicles and the ability to cross biological barriers for RNA delivery [137]; polymer-based nanocarriers encapsulate and protect RNA by designing biocompatible polymers and enhancing the efficiency of targeted delivery through surface modification [138]. 

### 6.3. Enhancing RNA-Based Early Diagnosis Strategies

Enhancing the RNA early diagnosis strategy involves optimizing various components of the process, from biomarker discovery and validation to assay development and clinical implementation. By utilizing advanced high-throughput sequencing techniques, such as single-cell RNA sequencing and spatial transcriptomics, RNA biomarkers with high sensitivity and specificity can be identified [139]. Through single-cell sequencing, Liulin et al. identified a close association between certain genes, including *SPG20* and *FRAT2*, and Alzheimer’s disease [140]. *NNMT*, *PTGS2*, etc., are closely related to ulcerative colitis [141]. Holly R. et al. utilized spatial transcriptomics to investigate lncRNAs in CRC tumors, identifying LINC01978, PLAC4, and LINC01303 as potential biomarkers for early risk assessment of metastatic disease [142]. Nanomaterial-assisted metabolic analysis has made great progress and has many applications in in vitro diagnostics [143]. Integrated omics approaches combine RNA sequencing data with other omics data (such as proteomics and metabolomics) to help identify comprehensive biomarker signatures and gain insight into disease mechanisms [144]. In addition, digital PCR and droplet digital PCR technologies are used for ultra-sensitive and precise quantitative analysis of low-abundance RNA, and multiplexed detection methods are developed to simultaneously detect multiple RNA biomarkers, thereby improving the accuracy and efficiency of diagnosis. Strengthening techniques for isolating and enriching exogenous RNA, along with other forms of extracellular RNA from body fluids, is essential to enhance both yield and purity. Artificial intelligence (AI) is revolutionizing the healthcare industry using technologies such as machine learning (ML), natural language processing (NLP), and computer vision. For instance, during the COVID-19 pandemic, physicians utilized AI to analyze CT imaging, enabling precise assessment of lesion volume and morphology [145]. Utilizing machine learning and AI algorithms to analyze RNA sequencing data can help identify patterns and accurately predict disease states. Meta-learning is an advanced AI paradigm designed to enhance learning efficiency on new tasks by analyzing and synthesizing knowledge gained from multiple prior tasks. Hong et al. developed a neural network-based meta-learning framework that efficiently analyzes sequencing data and delivers exceptional predictive performance on target cancer sites [146]. Additionally, combining RNA biomarker data with electronic health records and other clinical information can refine predictive models, enabling personalized diagnostics [131]. 

To improve early diagnosis strategies involving RNA, a comprehensive approach is needed. This should include advanced biomarker discovery, thorough validation, and the development of sensitive and specific assays, along with optimized sampling techniques. Integrating these elements with sophisticated data analytics, continuous monitoring, improved clinical implementation, and fostering collaboration and innovation can lead to more accurate, reliable, and accessible early disease diagnoses. Ultimately, these efforts will enhance patient outcomes and advance the field of precision medicine.

## 7. Conclusions

In conclusion, RNA-based diagnostics and therapeutics hold immense potential for transforming the management of gastrointestinal tumors. These advanced strategies offer unparalleled specificity and sensitivity, enabling early detection and precise targeting of tumor cells. By focusing on key RNA molecules such as miRNAs, lncRNAs, and circRNAs, it is possible to inhibit tumor metastasis and overcome drug resistance, significantly improving patient outcomes. Future directions include improving RNA targeting and integrating AI to improve therapeutic design and predict effective targets. The incorporation of RNA-based approaches into cancer care represents a significant shift in oncology, with ongoing research necessary to maximize their potential for effective clinical application.

## Figures and Tables

**Table 1:** RNA therapeutic applications in clinical trials.

Name	Type of RNA	Disease	Phase
BNT162b2	mRNA	COVID-19	FDA authorization for emergency use in 2020
mRNA-1273	mRNA	COVID-19	FDA authorization for emergency use in 2020
CVnCoV	mRNA	COVID-19	Phase III
AZD8601	mRNA	Ischemic heart disease	Phase II
mRNA-1647	mRNA	Cytomegalovirus infection	Phase II
P-BCMA-101	mRNA	Multiple myeloma	Phase II
mRNA-4157	mRNA	Cancer	Phase II
AGS-004	mRNA	HIV infections	Phase II
AGS-003-LNG	mRNA	Non-small-cell lung cancer	Phase II
iHIVARNA-01	mRNA	HIV infections	Phase II
AGS-003	mRNA	Renal cell carcinoma	Phase II
AZD8601	mRNA	Heart failure	Phase II
(MRG-106)	miRNA	Blood cancers	Phase II
(MRG-201)	miRNA	Keloids	Phase II
Inotersen (Tegsedi)	ASO	Familial amyloid polyneuropathy	FDA approval in 2018
IONIS-GCGR Rx	ASO	Type 2 diabetes	Phase II
ASM8	ASO	Allergen-induced asthma	Phase II
SB010	ASO	Asthma	Phase II
SB011	ASO	Atopic dermatitis	Phase II
G4460	ASO	Liquid cancer	Phase II
BP1001	ASO	Myeloid leukemia	Phase II
IONIS-FXI Rx	ASO	Clotting disorders	Phase II
STK-001	ASO	Dravet syndrome	Phase II
Eteplirsen (AVI-4658)	ASO	Duchenne muscular dystrophy	Phase III
Alicaforsen	ASO	Pouchitis	Phase III
IONIS-TTR Rx	ASO	Familial amyloid polyneuropathy	Phase III
Custirsen (OGX-011)	ASO	Prostate cancer	Phase III
Patisiran (Onpattro)	siRNA	Polyneuropathy	FDA approval in 2018
Givosiran (Givlaan)	siRNA	Acute hepatic porphyria	Phase II
siG12D-LODER	siRNA	Pancreatic cancer	Phase II
ALN-PCSSC	siRNA	Hypercholesterolemia	Phase II
PF-655	siRNA	Diabetic macular edema	Phase II
SYL1001	siRNA	Dry eye syndrome	Phase II
SYL040012	siRNA	Glaucoma	Phase II
QPI-1002	siRNA	Prevention of acute kidney injury	Phase II
PF-04523655	siRNA	CNV-AMD	Phase II
ALN-TTR02	siRNA	Familial amyloid polyneuropathy	Phase III
Pegaptanib (Macugen)	Aptamer (RNA)	Macular degeneration	FDA approval in 2014
Pegcetacoplan	Aptamer (RNA)	Geographic atrophy	Phase III
E10030	Aptamer (RNA)	Macular degeneration	Phase II
NOX-H94	Aptamer (RNA)	Anemia of chronic disease	Phase II
BT200	Aptamer (RNA)	Genetic	Phase II
Zimura	Aptamer (RNA)	Degeneration	Phase III

Clinical trial and approval data were collected from https://clinicaltrials.gov/, accessed in 1 December 2024.

## Data Availability

No new data were created or analyzed in this study. Data sharing is not applicable to this article.
